# Supplementary material for: Perceived stress may mediate the relationship between antenatal depressive symptoms and preterm birth: A pilot observational cohort study
Source: PLoS One. 2021 May 4;16(5):e0250982. doi: 10.1371/journal.pone.0250982 (PMC8096039; doi:10.1371/journal.pone.0250982)
Supplement: Attachment — Submitted filename: Responses to Reviewer Feedback.docx [file pone.0250982.s003.docx]

**Responses to Reviewer Feedback**

| **Reviewer Feedback** | **Responses** |
| --- | --- |
| In response to the sample size justification, authors indicated that the study involved secondary analysis. However, there is no mention about it in the manuscript. In-fact, authors have described sample recruitment and prospective design. I am not sure how this study is a secondary analysis. More information will be appreciated. | We thank the reviewer for this comment. We have added in the following sentence in the study design section: “This is a secondary analysis of data that were collected between October 2015 and July 2016.” |
| I am still not understanding the actual meaning of this sentence: “In addition, women who experienced consistently low or high depression scores did not have a higher odd of preterm birth as compared to women who experienced a decrease in depression scores.” I am not sure what authors are trying to convey? | We thank the reviewer for this comment. We have deleted the sentence in question to improve clarity and flow of the manuscript. |
| Tables can be improved per journal standard. | We thank the reviewer for this comment. We have taken the information below from the journal’s author guidelines and have reformatted our tables accordingly.  https://journals.plos.org/plosone/s/submission-guidelines  Cite tables in ascending numeric order upon first appearance in the manuscript file.  Place each table in your manuscript file directly after the paragraph in which it is first cited (read order). Do not submit your tables in separate files.  Tables require a label (e.g., “Table 1”) and brief descriptive title to be placed above the table. Place legends, footnotes, and other text below the table.  <https://journals.plos.org/plosone/s/tables>  Formatting Rules and Constraints   - **Tables must be editable, cell-based objects.** We cannot typeset tables that are graphic objects. Use the table tool in your text editing software to create tables. If necessary, create tables in Excel and insert them into the manuscript. Do not insert text boxes or graphics within your tables. - **Text justification.** Justification of the text within cells is honored at typesetting. If you have a preference, indicate justification of content within cells by using your text editor’s alignment features (for example, centered or left justified).   Do not use returns, spaces, or tabs to align content across columns. To have content appear on separate lines within the same cell, you can [insert a list](https://journals.plos.org/plosone/s/tables#loc-arrangement-of-cell-content).   - **Merged cells.** You can use merging to indicate cells that span multiple columns and rows. - **Multi-section tables.** Tables with multiple sections must have a consistent number of columns throughout all sub-sections. If necessary, you may divide and re-number the sections as separate tables. Do not insert tables within tables or cells within cells. - **Text color and formatting.** Text color is limited to black. Bolded, italicized, underlined, superscript, subscript, and strikethrough text is OK. Meaning can also be expressed with symbols that are explained in the footnotes. Text in header rows will be automatically formatted in bold type. - **Text Font.** Use a standard font size and any standard font, except for the font named “Symbol”. To add symbols to the manuscript, use the Insert → Symbol function in your word processor or paste in the appropriate Unicode character. - **Cell shading.** Background color can be applied to cells to convey meaningful information. [Read the instructions for applying cell shading](https://journals.plos.org/plosone/s/tables#loc-cell-shading). - **Size.** Tables do not have strict width and height requirements. Do not split your table or otherwise try to make the table appear within the manuscript margins if it does not fit on one page. In Word, tables that run off of the manuscript page can be seen using Draft View. In the PDF version of the published article, very wide tables may be printed sideways, and long tables may span more than one page.   **Aligning content across rows and columns**   - Enter content in separate cells to match the text alignment used in other rows and columns. - Do not use returns, spaces, or tabs to align content across the table. Alignment set in this manner may not be preserved in the published version of the article. |
