# Supplementary material for: Perceived stress may mediate the relationship between antenatal depressive symptoms and preterm birth: A pilot observational cohort study
Source: PLoS One. 2021 May 4;16(5):e0250982. doi: 10.1371/journal.pone.0250982 (PMC8096039; doi:10.1371/journal.pone.0250982)
Supplement: Attachment — Submitted filename: Reviewer comments on revised version.docx [file pone.0250982.s002.docx]

**Reviewer comments for authors**

Thank you for the opportunity to review the revised manuscript titled “Perceived stress may mediate the relationship between antenatal depressive symptoms and preterm birth: a pilot observational cohort study.” I am thankful that authors have attempted to address majority of the comments. However, there are a few places of ambiguity in the manuscript:

- In response to the sample size justification, authors indicated that the study involved secondary analysis. However, there is no mention about it in the manuscript. In-fact, authors have described sample recruitment and prospective design. I am not sure how this study is a secondary analysis. More information will be appreciated.
- Tables can be improved per journal standard.
- I am still not understanding the actual meaning of this sentence: “In addition, women who experienced consistently low or high depression scores did not have a higher odd of preterm birth as compared to women who experienced a decrease in depression scores.” I am not sure what authors are trying to convey?

Overall feedback: Minor revision
